# Estimation of the Fe and Cu Contents of the Surface Water in the Ebinur Lake Basin Based on LIBS and a Machine Learning Algorithm

**DOI:** 10.3390/ijerph15112390

**Published:** 2018-10-28

**Authors:** Xianlong Zhang, Fei Zhang, Hsiang-te Kung, Ping Shi, Ayinuer Yushanjiang, Shidan Zhu

**Affiliations:** 1 Key Laboratory of Smart City and Environmental of Higher Education Institute, College of Resources and Environment Sciences, Xinjiang University, Urumqi 830046, China; zhangxianlong3s@163.com (X.Z.); aynur226@163.com (A.Y.); 15276606619@163.com (S.Z.); 2 Key Laboratory of Oasis Ecology, Xinjiang University, Urumqi 830046, China; 3 Engineering Research Center of Central Asia Geoinformation Development and Utilization, National Administration of Surveying, Mapping and Geoinformation, Urumqi 830002, China; 4 Department of Earth Sciences, The University of Memphis, Memphis, TN 38152, USA; hkung@memphis.edu; 5 School of Foreign Language, Jining Medical University, Jining 272067, China; lottieshi@163.com

**Keywords:** laser-induced breakdown spectroscopy (LIBS), machine learning algorithm, Fe and Cu contents, estimation

## Abstract

Traditional technology for detecting heavy metals in water is time consuming and difficult and thus is not suitable for quantitative detection of large samples. Laser-induced breakdown spectroscopy (LIBS) can identify multi-state (such as solid, liquid, and gas) substances simultaneously, rapidly and remotely. In this study, water samples were collected from the Ebinur Lake Basin. The water samples were subjected to LIBS to extract the characteristic peaks of iron (Fe) and copper (Cu). Most of the quantitative analysis of LIBS rarely models and estimates the heavy metal contents in natural environments and cannot quickly determine the heavy metals in field water samples. This study creatively uses the Fe and Cu contents in water samples and the characteristics of their spectral curves in LIBS for regression modelling analysis and estimates their contents in an unknown water body by using LIBS technology and a machine learning algorithm, thus improving the detection rate. The results are as follows: (1) The Cu content of the Ebinur Lake Basin is generally higher than the Fe content, the highest Fe and Cu contents found within the basin are in the Ebinur Lake watershed, and the lowest are in the Jing River. (2) A number of peaks from each sample were found of the LIBS curve. The characteristic analysis lines of Fe and Cu were finally determined according to the intensities of the Fe and Cu characteristic lines, transition probabilities and high signal-to-background ratio (S/B). Their wavelengths were 396.3 and 324.7 nm, respectively. (3) The relative percent deviation (RPD) of the Fe content back-propagation (BP) network estimation model is 0.23, and the prediction ability is poor, so it is impossible to accurately predict the Fe content of samples. In the estimation model of BP network of Cu, the coefficient of determination (R^2^) is 0.8, the root mean squared error (RMSE) is 0.1, and the RPD is 1.79. This result indicates that the BP estimation model of Cu content has good accuracy and strong predictive ability and can accurately predict the Cu content in a sample. In summary, estimation based on LIBS improved the accuracy and efficiency of Fe and Cu content detection in water and provided new ideas and methods for the accurate estimation of Fe and Cu contents in water.

## 1. Introduction

Arid and semi-arid areas together constitute almost one-third of the world’s land area and half of China’s. Lakes in these areas are important inland ecosystems, as they function as vital water sources for local economic development and provide services ranging from food and drinking water to transportation and recreation [1,2]. With the rapid development of the oasis economy in recent years, climate change and human activities such as salinization, shrinkage of lakes, and serious pollution of heavy metals have threatened the hydrological environment and water quality of inland river basins in arid areas [3]. Owing to rapid development in economy and industry, heavy metal pollution is becoming more serious and has become one of the most important environmental problems in China. Therefore, the environmental pollution caused by heavy metals has aroused widespread concern [4].

To reduce environmental pollution and mitigate the resulting degradation of soil and water resources [5], heavy metal concentrations should be determined accurately. Various techniques have been established to detect heavy metal ions (HMIs), including inductively coupled plasma mass spectrometry (ICP-MS) [6], inductively coupled plasma optical emission spectrometry (ICP-OES) [7], inductively coupled plasma atomic emission spectrometry (ICP-AES) [8], flameless atomic absorption spectrophotometry (FAAS) [9] and atomic absorption spectroscopy (AAS) [10]. These are all highly sensitive and selective techniques, however, they require relatively expensive instruments, the application of complex operational procedures, and long detection times. Heavy metal pollution of oasis freshwater resources in arid areas has always been a highly important environmental problem worldwide. How to scientifically detect heavy metal content has become a research hotspot for relevant government departments, academics and environmentalists [11].

Laser-induced breakdown spectroscopy (LIBS) can test multi-state (such as solid, liquid and gas) substances simultaneously, rapidly and remotely [12,13]. The detected LIBS spectrum contains information about the constituents and the relative contents of the experimentally measured sample [14]. The types of elements in the sample can be identified by the wavelengths of the characteristic spectral lines, and the contents of these elements can be analysed by the relative intensities of the characteristic spectral lines [15]. Many scholars have used LIBS to conduct a large number of qualitative and quantitative water environmental pollution detection studies. Noll et al. (2001) used LIBS technology to detect the content of Ni in liquid steel and quickly monitor the quality of steel during the production process [16]. Schmidt et al. (2002) found that elemental analysis of solutions can be achieved by concentrating and immobilizing the metal ions into a commercially available ion exchange polymer membrane, followed by LIBS [17]. Gondal et al. (2007) used LIBS technology to detect more than a dozen kinds of harmful metals, such as lead, chromium, and zinc, in wastewater from dye factories [18]. Hussain et al. (2008) used LIBS technology to detect the plasma spectrum and analyse the contents of many toxic heavy metals in the wastewater from a dairy plant [19]. Järvinen et al. (2014) detected the heavy metal elements Ni, Pb and Zn in aqueous solution and created an online monitoring system by using LIBS for industrial wastewater [20]. Bhatt et al. (2017) utilized LIBS technology to quantitatively detect the heavy metal contents of europium and ytterbium in groundwater solutions [21]. The results of these studies have demonstrated that LIBS studies are more inclined to qualitatively and quantitatively analyse heavy metal elements in untested water, and inversion of the concentration of metal elements for untested water is rarely carried out.

Therefore, the purposes of this study are to: (1) use AAS to detect and analyse the contents of Fe and Cu in water samples from the Ebinur Lake Basin; (2) determine the characteristic peaks of Fe and Cu in the LIBS spectral curve; (3) use K-means clustering analysis and a back-propagation (BP) neural network and other machine learning algorithms to creatively conduct regression modelling analysis between measured values of Fe and Cu contents in water samples and the characteristic peaks of Fe and Cu in the LIBS spectral curve; and (4) improve the accuracy and efficiency of Fe and Cu content detection and provide a new method for accurately estimating and determining the Fe and Cu contents in the field.

## 2. Materials and Methods

### 2.1. Study Area

**Figure 1:**
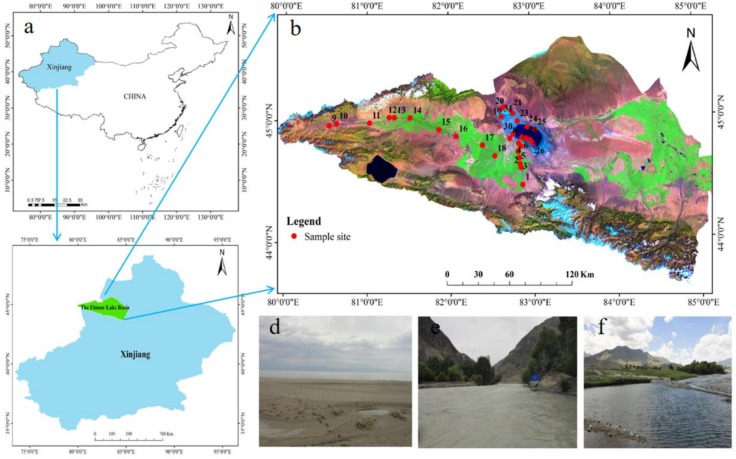
(**a**) Map of the locations of the Xinjiang Autonomous Region within China; (**b**) map of the study area in the Xinjiang Autonomous Region; (**c**) map of the research area and sampling points; (**d**) picture of Ebinur Lake; (**e**) picture of the Jing River; (**f**) Picture of Bortala River (photographed by Xianlong Zhang, Map by ArcGIS 10.2.2 (Environmental Systems Research Institute, RedLands, CA, USA).

The Ebinur Lake Basin is located in the northwest arid area in Xinjiang Uygur Autonomous Region, and it ranges from 44°54′ to 45°08′ N and 82°35′ to 83°10′ E (Figure 1) [22]. Ebinur Lake is a national desert natural ecological protection area and the lowest depression and saltwater-collecting centre in western of Junggar Basin [23]. The Bortala River, Jing River, and Kuitun River run into Ebinur Lake from the west, south, and east, respectively, and are the main sources of lake water [24]. The surface of the lake is elliptical, with an area of 650 km^2^. The average water depth is 2–3 m, and the lake surface is 189 m above sea level. The average annual temperature is 7.36 °C, the average annual precipitation is 100~200 mm, and the annual average evaporation is 1500~2000 mm [25]. The Ebinur Lake Basin is characterized by a lack of rainfall and dramatic temperature changes, which are typical of the arid continental climate of the north temperate zone [26].

### 2.2. Sample Collection and Laboratory Analysis

Water samples were collected from upstream to downstream at intervals of 4 km on Jing River and Bortala River, as well as in the artificially accessible areas around the Ebinur Lake district; these were taken On October 12 to October 19, 2017; then, a total of 31 water samples were collected and information on the latitude and longitude coordinates, terrain, geomorphology, and characteristics of the ecological environment were accurately recorded of each water sample. Finally, eight water samples were taken from the Bortala River, ten water samples were taken from the Jinghe River, and thirteen water samples were collected around the Ebinur Lake area. The water was sealed in a 1 L PVC bottle and stored at a low temperature.

Atomic absorption spectrometry has the advantages of low detection limit, high accuracy and little interference, reagent and resource saving and simple operation [27]. In order to establish an estimation model of Fe and Cu content in unknown water by using the characteristic peak of Fe and Cu in LIBS spectral curves. Therefore, after the samples were brought back to the laboratory, the contents of Fe and Cu in the water samples were determined using a TAS-990 atomic absorption spectrophotometer.

### 2.3. LIBS Experiment Device and Data Acquisition

**Figure 2:**
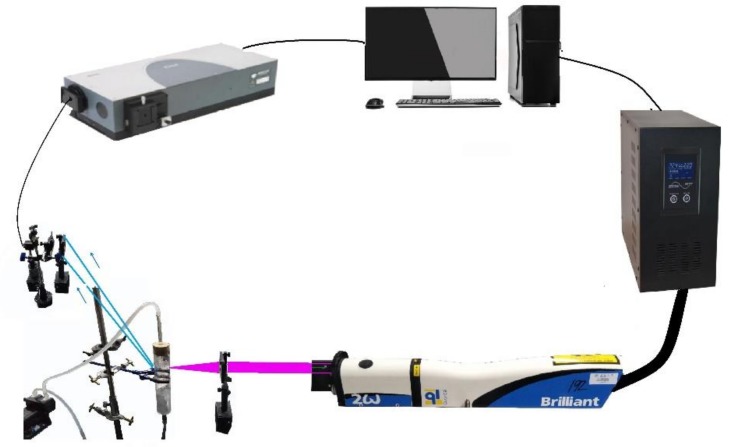
Schematic diagram of LIBS experimental device.

LIBS is a typical atomic emission spectrum, and its working principle is shown in Figure 2. The system mainly consists of a power supply, laser, spectrometer, computer, delay pulse generator, ancillary equipment, and other components [28].

The general auxiliary devices mainly included reflected mirrors, lenses, optical fibres, and liquid jet systems. In this paper, the Nd: YAG laser (Brilliant B) that is produced by the French company QUANTEL was used as the laser light source; its output wavelength is 532 nm, laser fluency is 6.0 mJ/cm^2^, pulse width is 5 ns. The Shamrock 500i spectrometer is produced by the British company ANDOR and is used to collect spectral information. The wavelength response range is 180–850 nm, and the optical resolution is approximately 0.04 nm. The water sample undergoes circulated flow in the fluidic system to prevent liquid splashing. The liquid fluidic system consists of a peristaltic pump, experimental stand, glass tube, and beaker [29]. The peristaltic pump is used to ensure the circulating flow of the liquid and regulate the jet velocity of the liquid. The liquid fluidic system is fixed on the experimental platform, and the laser beam is focused on the water column by changing the position of the filter [30].

When a laser pulse beam is produced, the laser is focused on the flowing water column in the fluidic system through mirrors and lenses. Under strong pulse energy, some materials from the water sample are instantly vaporized and form plasma-containing electrons, ions, and atoms through the process of re-excitation [31]. The light emitted by plasma is focused on the optical fibre through a lens or collection device. Then, the light is transmitted to the spectrometer, detected by the detector, and converted to an electrical signal [32]. In addition, the delay pulse generated by the delay controller is used to control the time of plasma spectral signal acquisition. Thus, bremsstrahlung produced by the measured object during the initial stage of plasma formation is avoided, and the best spectral information is obtained [33].

### 2.4. LIBS Data Preprocessing

**Figure 3:**
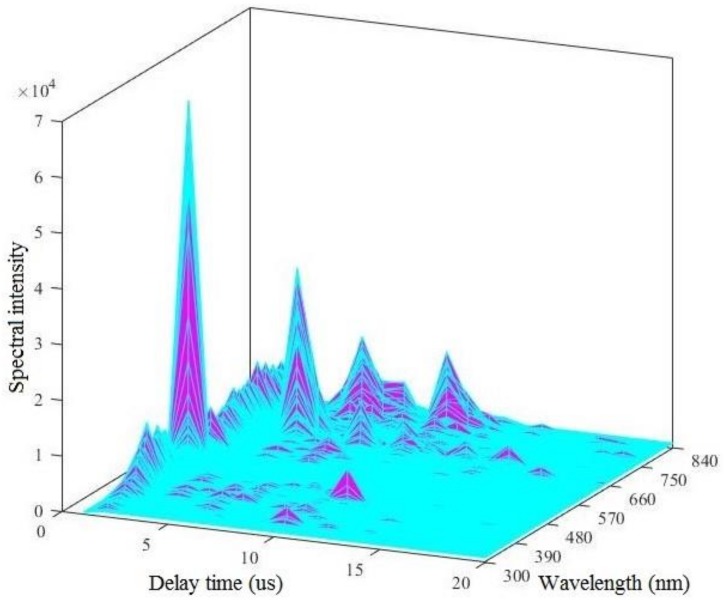
Three-dimensional LIBS spectra.

The original LIBS spectral signal contains a large amount of information that is unrelated to the sample composition, such as instrument noise, background and matrix interference [34], and this information causes serious baseline drift and a poor signal-to-noise ratio of the LIBS spectrum (Figure 3). Therefore, to improve the detection accuracy of the LIBS spectrum, the original data need to be preprocessed to reduce errors that are caused by improper operation or parameter setting errors during the test process after obtaining the LIBS spectral information from the water sample. In this paper, the pre-processing data from LIBS mainly includes abnormal value elimination, mean processing, baseline removal, and wavelet analysis [35].

#### 2.4.1. Baseline Correction Principle and Method

Due to the existence of blackbody radiation, bremsstrahlung radiation, load radiation and some molecular radiation, there are higher continuous background interference and baseline drift, which has a serious impact on the accurate extraction of characteristic spectral lines. Therefore, it is necessary to deduct the background spectral intensity to achieve baseline correction. In the LIBS quantitative analysis technique, baseline correction is an essential part of the LIBS data preprocessing. Baseline calibration is used to calibrate the peak position and peak intensity of the characteristic elements. At present, quite a few researchers mainly use polynomial fitting method to implement baseline correction of LIBS spectral curve. The implementation of baseline correction is shown in the following [36,37].

(1) First, the range of the LIBS band is divided into *N* groups, *W* is the wavelength range of the spectral data and *Q* is the number of data points for each group after they are equally distributed:

(1) $$Q=\frac{W}{N}\;$$

Second, the data in *N* group are processed separately, and the minimum spectral intensity of each group is selected as the eigenvalue:

(2) $$\min{\left\{A\left(\alpha_{i}\right)\right\}}_{j}\;j\;=\;1,2,3\ldots n\;$$

(2) The eigenvalue point in each set of data is connected, and spline interpolation is used to fit a curve to obtain the baseline function (f_baseline_). The difference between the original spectral data (spec_org_) and the baseline data (f_baseline_) is the corrected spectral data (spec_correct_):

(3) $$spec_{correct}\;=\;spec_{org}-f_{baseline}\;$$

#### 2.4.2. Wavelet Transform Principle and Method

There are many kinds of signal denoising methods, such as Kalman filter method, Wiener filter method, subtraction method, and so on. Because the wavelet transform algorithm has the advantages of good time-frequency localization, base selection flexibility and de-correlation, it can be used to distinguish high-frequency signals from noise components.

In mathematics, a wavelet series is a representation of a square-integrable function by a certain generated orthonormal series [38]. A wavelet is a wavelet-based or wavelet-generating function Ψ(t) that satisfies the condition and generates a family of functions Ψa,b (t) through scaling and translation [39]:

(4) $$\Psi a,b(t)=\frac{1}{\sqrt{\left|a\right|}}\Psi \left(\frac{t-b}{a}\right),a,b\in R,a\neq 0\;$$

Among them, *a* is the scale factor that is used to control scaling; ***b*** is the translation factor that is used to control translation; and Ψa,b(t) is the wavelet function of Ψ(t) after translation and expansion:

(5) $$WT_{x}(a,b)=\left(x(t),\Psi a,b\left(t\right)\right)=\frac{1}{\sqrt{\left|a\right|}}\int_{-\infty }^{+\infty }x(t)\Psi a,b\left(t\right)dt$$

The essence of WT is to project the signal x(t) onto the wavelet Ψa,b(t), which is the inner product of x(t) and Ψa,b(t), and obtain the wavelet coefficients, which are easy to address. The wavelet coefficients are processed according to the need of analysis, and the processed wavelet coefficients are then inverse transformed to obtain the processed signals. In practical applications, discrete wavelet transformation is usually used, and its discrete wavelet is defined as:

(6) $$a=a_{0}^{m},b=na_{0}^{m}b_{0},(m,n\in Z,a_{0}\neq 0)\;$$

The above formula can be converted into:

(7) $$\Psi_{m,n}(t)=\int_{-\infty }^{+\infty }x(t)a_{0}^{-\frac{m}{2}}\psi (a_{0}^{-m}t-nb_{0})\mathrm{dt}\;$$

For k discrete spectral data points (x1, x2, …, xk) with equal wavelength intervals, its discrete dyadic wavelet transform is:

(8) $$WT_{x}(m,n)=\left(x_{i},2^{-\frac{m}{2}}\Psi (2^{-m}t_{i}-n)\right)=\sum_{i=1}^{k}\Psi (2^{-m}t_{i}-n)x_{i}\;$$

The above formula shows that WT is actually a projection of a discrete signal on a wavelet basis function. Different m and n values represent different resolutions (scales) and time domains (translations), respectively. The wavelet function is generated through different m and n values to adjust the different local time domains and resolutions [40].

### 2.5. BP Neural Network Model

Machine learning is a method used to devise complex models and algorithms that lend themselves to learn from and make predictions on data. Machine learning algorithms mainly include decision tree learning, association rule learning, artificial neural networks, deep learning, support vector machines, genetic algorithms, Bayesian networks etc. Among them, A BP network is an effective learning method for multilayer neural networks [41]. BP network has self-learning, self-organization, self-adaptation, strong fault-tolerant, distributed storage, parallel processing of information and powerful nonlinear approximation abilities [42]. By constantly adjusting the network weight value, the final output of the network is as close as possible to the expected output, so it can achieve the purpose of training [43,44]. When the input sample has large error or individual error, it has little effect on the input and output rule of the network. In addition, the accuracy of the estimation model can be easily affected by the anomaly of the individual data when the amount of data in the modeling set is small. Therefore, the BP neural network algorithm is used to establish an estimation model of Fe and Cu content.

**Figure 4:**
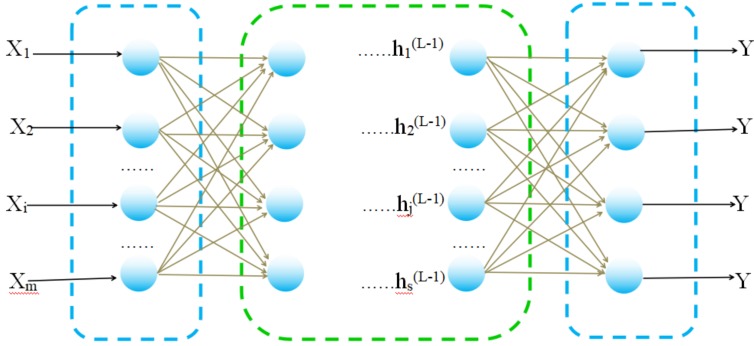
Schematic diagram of a BP neural network.

From the structure diagram of the neural network model shown in Figure 4, it can be seen that a multilayer neural network usually consists of L-layer neurons. The first layer is called the input layer, the last layer (the Lth layer) is called the output layer, and the other layers are called the hidden layers (the first layer to the *L-1* layer). The input vector is defined as [45,46]:

(9) $$\vec{\chi }=\left\{\chi_{1},\chi_{2}\ldots \chi_{i}\ldots \chi_{m}\right\},\;i\;=\;1,2,3,\ldots ,\;m\;$$

The output vector is defined as:

(10) $$\vec{y}=\left\{y_{1},y_{2}\ldots y_{k}\ldots y_{n}\right\},\;k\;=\;1,2,3,\ldots ,\;n\;$$

The output of each neuron in the first hidden layer is:

(11) $$h^{\left(l\right)}=\left[h_{1}^{\left(l\right)},h_{2}^{\left(l\right)}\ldots h_{j}^{\left(l\right)}\ldots h_{sl}^{\left(l\right)}\right],\;j\;=\;1,2,\ldots ,\;sl\;$$

where sl is the number of neurons in the first layer.

$net_{i}^{\left(l\right)}$ is defined as the connection weight between the j-th neuron in the L-1 layer and the i-th neuron in the L-th layer, and $b_{i}^{\left(l\right)}$ is defined as the offset of the i-th neuron in the L-th layer:

(12) $$h_{i}^{\left(l\right)}=f\left(net_{i}^{\left(l\right)}\right)\;$$

(13) $$net_{i}^{\left(l\right)}=\sum_{i=1}^{s_{l-1}}w_{ij}^{\left(l\right)}h_{j}^{\left(l-1\right)}+b_{i}^{\left(l\right)}\;$$

where $net_{i}^{\left(l\right)}$ is the input of the i-th neuron of the L-th layer, and f(·) is the activation function of the neuron.

Suppose we have m training samples{(x(1),y(1)), (x(2),y(2)), …, (x(m),y(m))} {(x(1),y(1)), (x(2),y(2)), …, (x(m),y(m))}, where d(i) is the expected output corresponding to the input x(i). For a given m training sample, the error function is defined as:

(14) $$E=\frac{1}{m}\sum_{i=1}^{m}E(i)\;$$

where E(i) is the training error for a single sample:

(15) $$E(i)=\frac{1}{2}\sum_{k=1}^{n}{\left(d_{k}(i)-y_{k}(i)\right)}^{2}\;$$

Therefore, the combination of Equations (14) and (15):

(16) $$E=\frac{1}{2m}\sum_{i=1}^{m}\sum_{k=1}^{n}{\left(d_{k}(i)-y_{k}(i)\right)}^{2}\;$$

The BP algorithm updates the weights and biases at each iteration in the following way:

(17) $$w_{ij}^{\left(l\right)}=w_{ij}^{\left(l\right)}-\alpha \frac{\partial E}{\partial W_{ij}^{\left(l\right)}}\;$$

(18) $$b_{i}^{\left(l\right)}=b_{i}^{\left(l\right)}-\alpha \frac{\partial E}{\partial b_{i}^{\left(l\right)}}\;$$

where α is the learning rate, and its range is (0, 1). The key to a BP algorithm is how to solve the partial derivative of $w_{ij}^{\left(l\right)}$ and $b_{i}^{\left(l\right)}$. For a single training sample, the process to calculate the weight of the partial derivative of the output layer is as follows:

(19) $$\frac{\partial E(i)}{\partial w_{kj}^{\left(L\right)}}=\frac{\partial }{\partial w_{kj}^{\left(L\right)}}\left(\frac{1}{2}\sum_{k=1}^{n}{\left(d_{k}(i)-y_{k}(i)\right)}^{2}\right)={-\left(d_{k}(i)-y_{k}(i)\right)f{\left(\chi \right)}^{\prime }|}_{\chi =net_{k}^{\left(L\right)}}h_{j}^{\left(L-1\right)}\;$$

(20) $$\frac{\partial E(i)}{\partial b_{k}^{\left(L\right)}}={-\left(d_{k}(i)-y_{k}(i)\right)f{\left(\chi \right)}^{\prime }|}_{\chi =net_{k}^{\left(L\right)}}\;$$

### 2.6. K-Means Clustering Method

Cluster analysis or clustering is the task of grouping a set of objects in such a way that objects in the same group are more similar to each other than to those in other groups [47]. Cluster analysis can be achieved by various algorithms that differ significantly in their understanding of what constitutes a cluster and how to efficiently find them. Popular notions of clusters include groups with small distances between cluster members, dense areas of the data space, intervals or particular statistical distributions. Clustering can therefore be formulated as a multi-objective optimization problem [48].

K-means clustering is a method of vector quantization, originally from signal processing, that is popular for cluster analysis in data mining. k-means clustering aims to partition n observations into k clusters in which each observation belongs to the cluster with the nearest mean, serving as a prototype of the cluster [49].

Given a set of observations (x_1_, x_2_, …, x_n_), where each observation is a d-dimensional real vector, k-means clustering aims to partition the n observations into k(≤n) sets S = {S_1_, S_2_, …, S_k_} so as to minimize the within-cluster sum of squares (WCSS). Formally, the objective is to find:

(21) $$\arg_{S}\min\sum_{i=1}^{k}\sum_{x\in S_{i}}{\left\|x-\mu_{i}\right\|}^{2}=\arg_{S}\min\sum_{i=1}^{k}\left|S_{i}\right|VarS_{i}\;$$

where μ_i_ is the mean of points in S_i_. This is equivalent to minimizing the pairwise squared deviations of points in the same cluster:

(22) $$\arg_{S}\min\sum_{i=1}^{k}\frac{1}{2\left|S_{i}\right|}\sum_{x,y\in S_{i}}{\left\|x-y\right\|}^{2}\;$$

The equivalence can be deduced from identity:

(23) $$\sum_{x\in S_{i}}{\left\|x-\mu_{i}\right\|}^{2}=\sum_{x\neq y\in S_{i}}\left(x-\mu_{i}\right)\left(\mu_{i}-y\right)\;$$

Because the total variance is constant, this is also equivalent to maximizing the sum of squared deviations between points in different clusters, which follows easily from the law of total variance. In this paper, the purpose of cluster analysis is to classify the study samples according to the different characteristics of the objects. The samples are gradually aggregated by the similarity of their attributes, and the highest similarities are first aggregated together. Finally, the sample is divided into a modeling set and a validation set by comprehensive features to reduce the impact of artificial random selection on the sample data.

### 2.7. Model Accuracy Test Method

The accuracy of the model is comprehensively used to measure the estimated ability of a model, the higher accuracy it is, the better in stability and the higher in verification accuracy; the prediction ability is also stronger. The modelling accuracy and estimation ability are mainly evaluated by the following parameters:

(1) Coefficient of determination (R^2^)

R^2^ is mainly used to evaluate the fitting degree of a regression model to a real model to measure the accuracy and stability of the model. The formula is as follows:

(24) $$R^{2}={\left(\frac{\sum_{i=1}^{n}(X_{i}-\bar{X})\left(Y_{i}-\bar{Y}\right)}{\sqrt{\sum_{i=1}^{n}{\left(X_{i}-\bar{X}\right)}^{2}}\sqrt{\sum_{i=1}^{n}{\left(Y_{i}-\bar{Y}\right)}^{2}}}\right)}^{2}\;$$

In the formula, Xi is the predicted value, $\bar{X}$ is the average of the predicted values, Yi is the measured value, $\bar{Y}$ is the average of the measured values, and *n* is the number of samples. The value range of R^2^ is 0 ≤ R^2^ ≤1, and if the value of R^2^ is closer to 1, it indicates that the fitting degree of the model to the data is better.

(2) Root mean square error (RMSE)

RMSE is the result of the root mean square of error of the sum of the predicted and measured values and is mainly used to evaluate the inversion capability of the model. The RMSE of the model sample is used to evaluate the modelling capability, and the RMSE of the prediction sample is used to evaluate the predictive ability. The formula is as follows:

(25) $$RMSE=\sqrt{\frac{1}{n}\sum_{i=1}^{n}{\left({Χ}_{i}-Y_{i}\right)}^{2}}\;$$

In the formula, Xi is the predicted value, Yi is the measured value, and n is the number of samples. The smaller the RMSE is, the higher the precision of the model and the stronger the inversion ability.

(3) Standard deviation (SD)

As the representative of random error, the SD is the statistical mean of the random error of the absolute value, which reflects the degree of dispersion among individuals in the group. The greater the SD of the sample, the greater the fluctuation of the sample data. The formula is as follows:

(26) $$SD=\sqrt{\frac{1}{N}\sum_{i=1}^{N}{\left(x_{i}-\mu \right)}^{2}}\;$$

In the formula, Xi is the random sample value, µ is the arithmetic average of the total sample value, and N is the total number of samples.

(4) Relative percent deviation (RPD)

The value obtained by dividing the SD from RMSE is the RPD, which is used to evaluate the accuracy of the model verification. The formula is as follows:

(27) $$RPD=\frac{SD}{RMSE}\;$$

The larger the RPD, the higher the accuracy of the model. When RPD > 2, it indicates that the model has excellent predictive ability; when 1.4 < RPD < 2, it indicates that the model can roughly estimate the sample; and when RPD < 1.4, it indicates that the model cannot accurately predict the sample [50].

## 3. Results and Analysis

### 3.1. Statistical Analysis of Fe and Cu Contents

**Figure 5:**
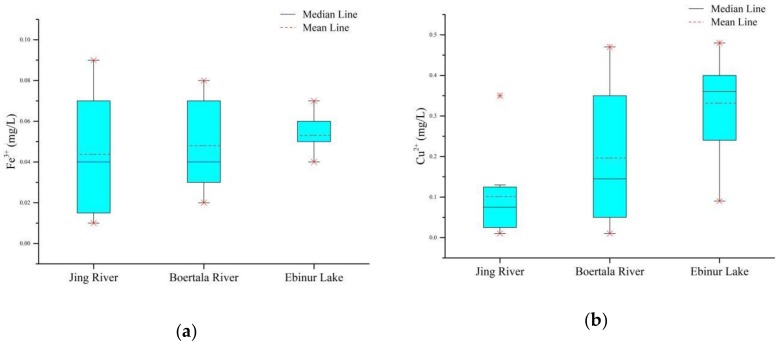
Box plots of (**a**) Fe and (**b**) Cu contents.

After water quality was determined, the data would be recorded, and a box plot of Fe and Cu contents would also be established, as shown in Figure 5. From the box plot of the contents of Fe and Cu, it can be found that the content of Cu in the Ebinur Lake Basin is higher than that of Fe in general. The average contents of Fe and Cu were highest in the Ebinur Lake, and the contents of Fe and Cu were lowest in the Jing River. The box plot of the content of Fe indicates that there are great differences in Fe content among different sampling points in the Jing River; the difference of Fe content at different sampling points in Ebinur Lake is small. The box plot of the content of Cu indicates that the content of Cu in the Jing River is relatively low, and the difference of Cu content at different sampling points is small. There are great differences in Cu content among different sampling points in the Bortala River, and the average value of the Cu content is the largest in Ebinur Lake.

**Figure 6:**
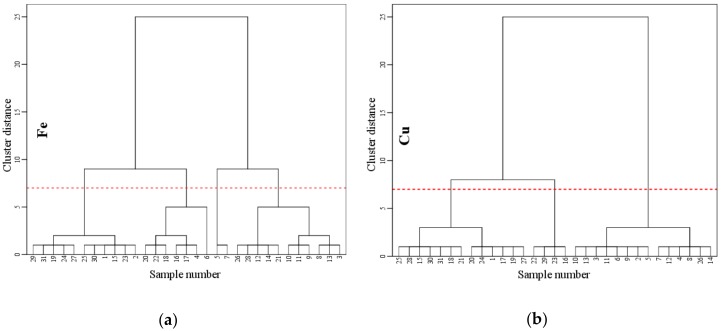
System cluster pedigree chart: (**a**) Fe; (**b**) Cu.

When establishing Fe and Cu content prediction models and testing the precision, it is often necessary to divide the measured samples into a modelling set and verification set. The modelling and verification sets are usually randomly selected from the sample data without considering the differences between the samples, which often leads to poor representativeness. In addition, the random selection of sample data may be influenced by humans, which can eventually change the accuracy of the model verification, seriously affect the reliability of the prediction results, and limit the applicability of the model [51]. According to the different characteristics of the objects, cluster analysis classifies the samples of study. The samples are gradually aggregated by the degrees of similarity of their properties, and the highest degree of similarity is aggregated together first. Finally, the samples are divided into several varieties by the comprehensive characteristics, and the entire process of the cluster analysis is completed. In this experiment, hierarchical cluster analysis was carried out according to the measured Fe and Cu contents, which were based on 31 water samples, as shown in Figure 6. On the basis of systematic clustering results, the K-means clustering method is applied for the final clustering analysis, and the sample data are classified into modelling and validation sets through the classification results.

According to the results of the hierarchical cluster analysis of Fe and Cu contents, the K-means clustering analysis of Fe content was divided into four categories, and the maximum number of iterations was ten. The K-means clustering analysis of the Cu content is divided into three categories, and the maximum number of iterations was ten. In each sample, the distances between the contents of Fe and Cu and the cluster centre are obtained; the results are shown in Table 1.

In the establishment and verification of the Fe and Cu content estimation model, the original measured data are usually divided into a modelling set and a verification set according to the ratio of 3:1. Through the classification results in the above table, the points with smaller cluster distances are selected as the verification set in each category to better reflect the water quality characteristics in each category. Therefore, the selection of the verification set is more general and applicable, which makes verification of the precision of the estimation model more scientific.

In the K-means clustering table of Fe, it can be found that in the first type of sample, the cluster distances of points #2, #15, #25, and #30 are smaller; in the second type of sample, the cluster distance of point #8 is smaller; in the third type of sample, the cluster distances of points #12 and #21 are smaller; and in the fourth type of sample, the cluster distance of point #17 is smaller. Therefore, these eight samples (#2, #15, #25, #30, #8, #12, #21 and #17) are selected as the verification set for the Fe estimation model. Similarly, eight samples (#15, #19, #25, #28, #3, #10, #13 and #22) are finally selected as the verification set for the Cu content estimation model in the K-means clustering table of Cu. The statistical characteristics of the modelling and the verification sets of the Fe and Cu content estimation models are shown in Table 2.

### 3.2. LIBS Spectral Characteristics of Water Samples

This paper first queried the Atomic Spectra Database (ASD) of the National Institute of Standards and Technology (NIST) and the standard curve of the Kurucz database. By comparing the wavelength of the peaks of LIBS curve with that of the corresponding elements in the database, it can be preliminarily judged that the characteristic spectral peaks of O and H elements are 777 nm and 656 nm respectively, as shown in Table 3. In addition, since the water samples to be tested and the air mainly contain O and H elements, the peak values of the characteristic bands of O and H elements are very large in the LIBS curve. Therefore the LIBS spectral intensity is extremely high at the 777 nm and 656 nm bands, which reduces the relative spectral intensities of the other bands. In this paper, the Nd: YAG laser (Brilliant B) was used as the laser light source; its output wavelength is 532 nm, pulse width is 5 ns. In the initial stage of plasma produced by the interaction of laser pulse with water, the spectrum radiated by the transition from free electrons to free states and from free states to bound states in the plasma. In the spectrum, the response is that there is a continuous background radiation at 532 nm and with a large noise [52]. Through the original three-dimensional (3D) LIBS spectrum of the water samples in Figure 3, it can be found that the noise interference is smaller in the range of the 300–500 nm band, the intensity of LIBS spectrum is obviously enhanced, and the background interference is larger in the range of the 500–800 nm band. In addition, by comparing the Atomic Spectra Database (ASD) of the National Institute of Standards and Technology (NIST), the characteristic peaks of Fe and Cu are mainly concentrated at 300–500 nm. To sum up, after pretreatment, the LIBS spectral intensity of the 300–500 nm band was selected as the research object, and the LIBS spectral curves of each sample were obtained.

**Figure 7:**
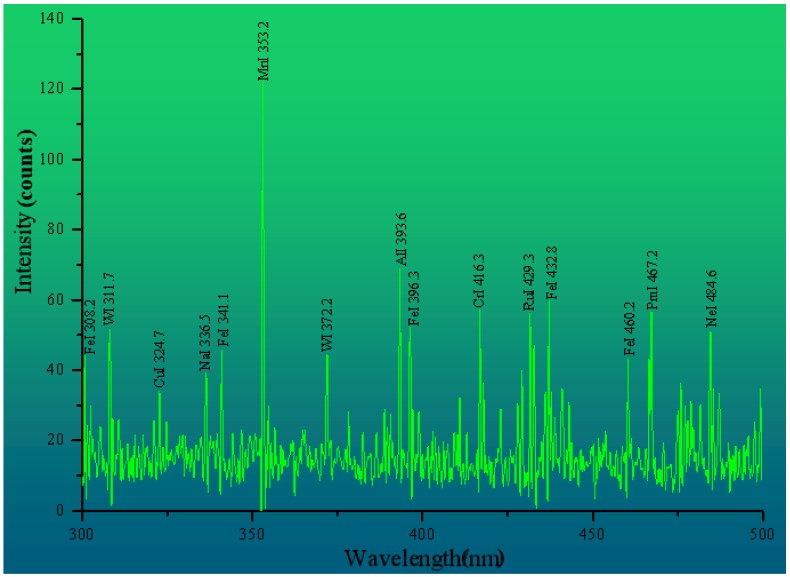
LIBS spectral curve of water sample #16 after pretreatment.

The measured values of the Fe and Cu contents in each water sample shows that the contents of Fe and Cu are lowest in water sample #16. In order to find out whether the LIBS detection limits and characteristic spectral lines of Fe and Cu can be detected by LIBS technique in all samples, this paper firstly carries out experimental operation on No. 16 water sample with the lowest concentration of Fe and Cu in all water samples, and tries to explore the characteristic peaks of Fe and Cu in LIBS spectral curve, as shown in Figure 7. From the results of Figure 7, it can be found that a plurality of characteristic bands of Fe and Cu can be detected in the LIBS spectral curve in the 300–500 nm range. Then all the water samples were subjected to LIBS experiments in turn and the characteristic spectral curves of each water sample were recorded in detail, which provided the data foundation for the later quantitative analysis.

During the quantitative analysis of the actual samples, it is necessary to identify the elements corresponding to the peaks of the spectral curve, which are the basis of the quantitative analysis. To obtain a higher signal-to-background ratio (S/B), the characteristic peaks are usually selected from a band with a higher peak value and no obvious interference spectra in the vicinity to improve the recognition rate of the characteristic peaks of the element. Therefore, the selection is the prerequisite to obtaining the information of the element type and the mass fraction in the sample. This paper queries the Atomic Spectra Database (ASD) of the National Institute of Standards and Technology (NIST); comprehensively considers factors such as the structure, intensity, excitation potential, and transition probability of each spectral line; and tries to determine the characteristic peaks of the elements to be tested with less interference from overlapping peaks. The characteristic peaks of Fe and Cu are found in the range of the 300–500 nm bands, as shown in Table 3.

**Figure 8:**
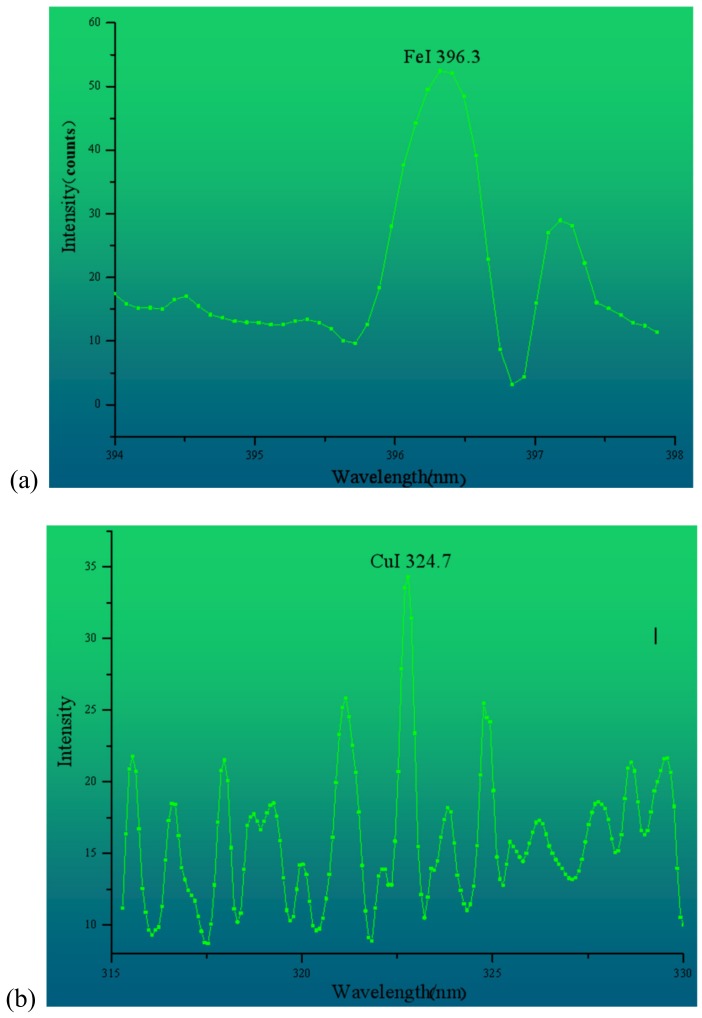
Magnification of characteristic peaks of (**a**) Fe and (**b**) Cu.

Considering the peak strength of Fe and Cu and the transition probability in each water sample, the characteristic peaks of Fe and Cu are finally determined as wavelengths of 396.3 nm and 324.7 nm, respectively. Figure 8 shows an enlarged view of the characteristic peaks of Fe and Cu in water sample #16. It can be seen that the structure is clear and almost undisturbed by the overlapping peaks. This peak can be used as the independent variable of the quantitative inversion model of Fe and Cu content to explore and improve the precision of the new method for estimating the water quality index.

### 3.3. Establishment and Accuracy Test of the Estimation Model

In the data analysis of LIBS, the intensity information of characteristic band of the sample to be measured is used to determine the content of its constituent elements by quantitative analysis method. The line intensity of characteristic band is directly proportional to the content of elements in the sample [53]. In practice, there are many characteristic spectral lines corresponding to the elements in the LIBS spectral curve. Affected by the drift of the center of the spectral line and the broadening of the spectral line, the peak area of the analysis of the characteristic spectral line may not be able to express its true intensity. In order to improve the accuracy of LIBS quantitative analysis, the intensity of the laser-induced plasma emission lines (i.e., the peak height of the lines) and the concentration of the corresponding elements were fitted to estimate the concentration of heavy metals in the water samples.

Using programming analysis in MATLAB 2014a software (The MathWorks, Natick, MA, USA), the characteristic peak strength of Fe at the 396.3 nm band in the LIBS spectral curve of each water sample is taken as the input variable. The Fe content of the 23 water samples in the modeling set is then measured as the output variable, and a BP neural estimation model is established for Fe content. In the same way, the characteristic peak strength of Cu at the 324.7 nm band is taken as the input variable, and the Cu content of each water sample is measured as the output variables to establish a BP neural network estimation model for Cu content. The results are shown in Table 4.

In the BP neural network, the hidden layer selects the logsig-moid type function, and the output layer selects the pureline type linear neuron so that the output of the entire network can be taken as an arbitrary value. The training function selects the trainlm to forward train the network by the Levenberg-Marquardt rule. The maximum number of model iterations is 1000, the allowable error is 0.001, the smoothing factor is 0.1, the penalty factor is 0.001, the initial learning rate is 0.5, and the maximum learning frequency is 5000 times. If the learning frequency is greater than 5000 times and the limit of the learning error is still not reached, then the model needs to start running again.

From the results in Table 4, it is found that the R^2^ of the Fe content estimation model is 0.89, the RMSE is 0.82, and the RPD is 7.93, and the R^2^ of the Cu content estimation model is 0.82, the RMSE is 0.4, and the RPD is 20.48. Overall, the RMSE of the Cu content estimation model is smaller than that of the Fe content estimation model; the RPD of the Cu content estimation model is much higher than that of the Fe content estimation model. This result shows that the Cu content estimation model based on the BP neural network has high precision and excellent prediction ability.

**Figure 9:**
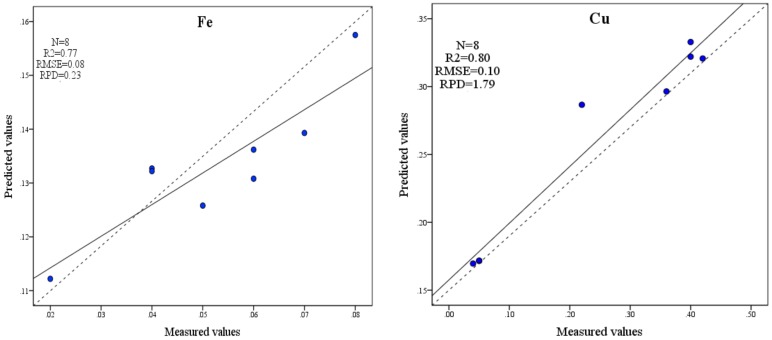
Accuracy test of BP neural network regression model.

To further verify the accuracy of the estimation model of Fe and Cu contents, a regression model inspection diagram between the predictive value of the model estimates and the measured values of Fe and Cu contents needs to be established, as shown in Figure 9. In the graph, the dotted line represents the 1:1 standard reference line and the solid line represents the linear fitting line. In the accuracy inspection chart of the estimation model of Fe content, the R^2^ is 0.77, the RMSE is 0.08, and the overall accuracy is better. However, the RPD is 0.23 and less than 1.4; it indicates that this model has poor predictive power and cannot accurately predict the samples.

In the accuracy inspection chart of the estimation model for Cu content, the R^2^ is 0.8, the RMSE is 0.1, the 1:1 standard reference line is close to the linear fit line of the sample point, and the deviation is small. In addition, the RPD is 1.79 and more than 1.4, which indicates that the BP neural network estimation model of Cu content has good accuracy and strong predictive ability, and it can accurately predict the Cu content in the sample.

## 4. Discussion

### 4.1. The Innovation of Fe and Cu Content Estimation

To improve the repeatability and accuracy of the LIBS quantitative analysis, some scholars have combined LIBS technology with chemical measurement methods [54]. Through these studies, it can be found that most of the quantitative analysis of LIBS rarely models and estimates the heavy metal contents in the natural environments and cannot quickly invert the heavy metals in the field water samples.

This research creatively uses the contents of Fe and Cu in water samples, which are measured by an atomic absorption spectrophotometer, and the characteristic curves of Fe and Cu in LIBS for regression modelling are analysed to give Fe and Cu content estimation models. Therefore, the contents of Fe and Cu in an unknown water body can be estimated by using LIBS technology, and the rate of water quality detection can be improved.

### 4.2. The Shortages of LIBS Data Acquisition and Processing

There are still some problems and difficulties in the LIBS experiment and post-processing operations that need further study. In arid areas, the contents of heavy metals in inland lakes are relatively low, and they are categorized as trace elements [55]. The intensities of element characteristic lines in the LIBS of the water are weak and cannot be easily measured. When excitation radiation generates the characteristic spectrum, there is a relatively strong and continuous background. At the same time, the spectral lines in LIBS are affected by the combined effects of various broadening mechanisms, resulting in a poor resolution of the elemental spectrum [56]. The use of conventional quantitative methods to detect the contents of heavy metals in water may cause large measurement errors. In addition, fluctuations in the experimental system parameters (lens-to-sample [57], detection angle [58], laser pulse energy [59], gas composition and pressure [60]) might result in changes in the instability of the LIBS signal. The problem with instability in LIBS data can be solved to a certain extent by the average of multi-pulsed LIBS data, but how to improve the stability of the spectral signal is still a problem in quantitative detection.

### 4.3. The Applicability of the Estimation Model

BP neural networks have strong predictive functions, which help weaken the influence of the matrix effect [61]. Sirven et al. (2014) contrasted the traditional quantitative analysis method, the PLSR method, with a BP neural network combined with LIBS technology to detect the content of Cr in soil [62]. The results showed that the BP neural network estimated model is more accurate. Therefore, the BP neural network estimation model of heavy metal content, which is based on LIBS technology, has important application value and prospects. In addition, there is currently little research on inversion of the contents of heavy metals in water samples from semi-arid areas using the LIBS technique. Therefore, this study can provide some reference for detecting the contents of heavy metals in water samples by the LIBS technique.

The ecological environment of the Ebinur Lake wetland reserve is extremely fragile. The heavy metal content in the Ebinur Lake area can be used as one of ecological environmental evaluation factors. The Fe and Cu content is a typical water contaminated heavy metal, reflecting the heavy metal content in the Ebinur Lake basin at a certain level. It is of great significance to monitor environmental pollution and for that reason Ebinur Lake was chosen as the research area to measure the content of Fe and Cu. However, this research is limited to a study in the Ebinur Lake Basin in an arid/semi-arid area. The precision of Fe and Cu content estimation models based on LIBS spectral characteristic peaks needs further research and demonstration. In addition, the Fe and Cu content estimation models are not universal, and the application and promotion in other regions still need further exploration.

## 5. Conclusions

This study creatively uses the Fe and Cu contents in water samples and the characteristics of their spectral curves in LIBS for regression modelling analysis through a K-means cluster analysis, a BP neural network and other machine learning algorithms. And estimates the Fe and Cu contents using models with the goal of being able to estimate their contents in an unknown water body by using LIBS technology, thus improving the detection rate. This study found the following:

(1) The content of Cu in the Ebinur Lake Basin is higher than that of Fe in general. The average contents of Fe and Cu were highest in Ebinur Lake, and the contents of Fe and Cu were lowest in the Jing River.
(2) A number of peaks were found from the LIBS curve. The characteristic analysis lines of Fe and Cu were finally determined according to factors such as intensities of the characteristic lines for Fe and Cu, transition probability and high S/B. Their wavelengths were 396.3 and 324.7 nm, respectively.
(3) The RPD of the Fe content BP neural network estimation model is 0.23, and the prediction ability is poor; thus, it is impossible to accurately predict the Fe contents of a sample. In the BP neural network estimation model of Cu content, the R^2^ is 0.8, the RMSE is 0.1 and the RPD is 1.79. This result indicates that the BP neural network estimation model of Cu content has good accuracy and strong predictive ability and can accurately predict the Cu content in the sample.

The research underlines that the proposed method improves the accuracy and efficiency of quantitative measures of Cu and Fe in water by LIBS, but the method results are not comparable with those of other classical LIBS data processing methods, which are calibration curve and calibration free, therefore, it is necessary to perform further comparative research explorations in the future.

## Figures and Tables

**Table 1:** The distance from Fe and Cu contents in each sample to the cluster centre.

Ion Species	Sample No.	Clustering Category	Cluster Distance	Sample No.	Clustering Category	Cluster Distance	Sample No.	Clustering Category	Cluster Distance
Fe	#1	1	0.00143	#29	1	0.00857	#11	3	0.00625
	**#2**	1	0.00143	**#30**	1	0.00143	**#12**	3	0.00375
	**#15**	1	0.00143	#31	1	0.00857	#14	3	0.00375
	#18	1	0.01143	#3	2	0.004	**#21**	3	0.00375
	#19	1	0.00857	#5	2	0.006	#26	3	0.00375
	#20	1	0.01143	#7	2	0.006	#28	3	0.00375
	#22	1	0.01143	**#8**	2	0.004	#4	4	0.0025
	#23	1	0.00143	#13	2	0.004	#6	4	0.0075
	#24	1	0.00857	#9	3	0.00625	#16	4	0.0025
	**#25**	1	0.00143	#10	3	0.00625	**#17**	4	0.0025
	#27	1	0.00857						
Cu	#1	1	0.04538	#30	1	0.04462	**#10**	2	0.01385
	**#15**	1	0.02462	#31	1	0.05462	#11	2	0.02385
	#17	1	0.04538	#2	2	0.03385	#12	2	0.06615
	#18	1	0.07462	**#3**	2	0.02385	**#13**	2	0.01385
	**#19**	1	0.03538	#4	2	0.05615	#26	2	0.02615
	#20	1	0.07538	#5	2	0.04385	#14	3	0.062
	#21	1	0.08462	#6	2	0.05385	#16	3	0.058
	#24	1	0.06538	#7	2	0.06615	**#22**	3	0.002
	**#25**	1	0.00462	#8	2	0.04615	#23	3	0.018
	#27	1	0.02538	#9	2	0.05385	#29	3	0.012
	**#28**	1	0.00462						

The bold sample numbers with the underline are selected as the verification set for estimation model.

**Table 2:** Data statistics of the Fe and Cu content estimation models.

Ion Species	Model	Sample Size	Minimum (mg/L)	Maximum (mg/L)	Mean (mg/L)	Standard Deviation	Variance/%
Fe	Estimation	23	0.01	0.09	0.05	0.02	47.16
	Verification	8	0.02	0.08	0.05	0.02	34.9
Cu	Estimation	23	0.01	0.48	0.22	0.16	71.97
	Verification	8	0.04	0.42	0.24	0.17	71.52

**Table 3:** The characteristic peaks of the main element.

Elements	Wavelength (nm)	Transition		Rel. Int.
		Upper Level	Lower Level	
OΙ	777.2	2s^22^p^3^(^4^s°)3p	2s^2^2p^3^(^4^s°)3s	870
H	656.3	3p 2P° 1/2	2s 2S 1/2	500000
FeΙ	308.2	3d^6^(^3^F2)4s4p(^3^P°)	3d^7^(^4^F)4s	1
FeΙ	341.1	3d^6^(^3^G)4s4p(^3^P°)	3d^7^(^2^G)4s	1550
FeΙ	396.3	3d^6^(^5^D)4s(^6^D)4d	3d^6^(^5^D)4s4p(^3^P°)	5500
FeΙ	460.2	3d^7^(^4^F)4p	3d^7^(^4^F)4s	760
CuΙ	324.7	3d^10^4p	3d^10^4s	10000r
CuΙ	327.4	3d^10^4p	3d^10^4s	10000r

**Table 4:** The results of Fe and Cu content estimation models.

Estimation Model of Elements	R^2^	RMSE	SD	RPD
Fe	0.89	0.82	6.51	7.93
Cu	0.82	0.40	8.28	20.48
